# Spatiotemporal Medicare Reimbursement Trends and Economic Clusters in Otologic/Neurotologic Surgery

**DOI:** 10.1002/lary.70652

**Published:** 2026-06-05

**Authors:** Akshay Warrier, Shahid Iqbal, Daniel Bu, Ryan Bartholomew, Daniel J. Lee

**Affiliations:** ^1^ Department of Otolaryngology‐Head and Neck Surgery Rutgers New Jersey Medical School Newark New Jersey USA; ^2^ Department of Otolaryngology‐Head and Neck Surgery, Massachusetts Eye and Ear Infirmary Harvard Medical School Boston Massachusetts USA; ^3^ Department of Otolaryngology‐Head & Neck Surgery University of California San Diego La Jolla California USA

**Keywords:** geographic variation, healthcare economics, Medicare reimbursement, otology, procedural clustering, value‐based care

## Abstract

**Objectives:**

To characterize national trends in Medicare reimbursement for otologic/neurotologic procedures from 2012 to 2024, quantify the relative contributions of service volume, procedural mix, and within‐code reimbursement to aggregate spending, and identify clusters of procedures with similar economic behavior.

**Methods:**

Medicare Part B claims data from 2012 to 2024 were utilized and financial data were inflation‐adjusted to 2024 USD. National trends in volume, spending, and reimbursement per service were evaluated. A Shapley decomposition framework quantified the contributions of service volume, procedural mix, and within‐code reimbursement to changes in aggregate spending. Unsupervised clustering with principal component analysis, hierarchical clustering, and k‐means classified CPT codes by reimbursement growth, variability, and service volume. Geographic and rural–urban reimbursement variation were also assessed.

**Results:**

Annual otologic/neurotologic procedural volume increased from 1.85 million to 2.15 million services, while inflation‐adjusted mean reimbursement per service declined from $80.47 to $57.07. Total inflation‐adjusted spending fell from $147.1 million in 2012 to $121.9 million in 2024. Decomposition showed that rising service volume increased spending by $20.0 million, but this was offset by adverse shifts in procedural mix (−$7.7 million) and declining within‐code reimbursement (−$37.5 million). Clustering identified three economic phenotypes: low‐volume stable codes, high‐volume moderate‐variability codes, and high‐cost volatile codes. Geographic analysis demonstrated widening interstate and rural–urban reimbursement variation over time.

**Conclusion:**

Despite growing demand for otologic/neurotologic services, inflation‐adjusted Medicare reimbursement has steadily declined. Aggregate spending reductions were driven primarily by reimbursement erosion rather than reduced utilization, highlighting structural vulnerabilities in current fee‐schedule valuation for otologic/neurotologic care.

**Level of Evidence:**

III.

## Graphical abstract

Despite a 16.2% increase in Medicare‐reimbursed otologic/neurotologic procedural volume from 2012 to 2024, inflation‐adjusted reimbursement per service declined by 29.1%, resulting in reduced aggregate spending over time. Shapley decomposition demonstrated that reimbursement erosion, rather than decreased utilization, was the primary driver of spending decline, while unsupervised clustering identified distinct economic phenotypes among otologic/neurotologic CPT codes. These findings highlight structural vulnerabilities in current Medicare fee‐schedule valuation and growing geographic disparities in otologic/neurotologic reimbursement.

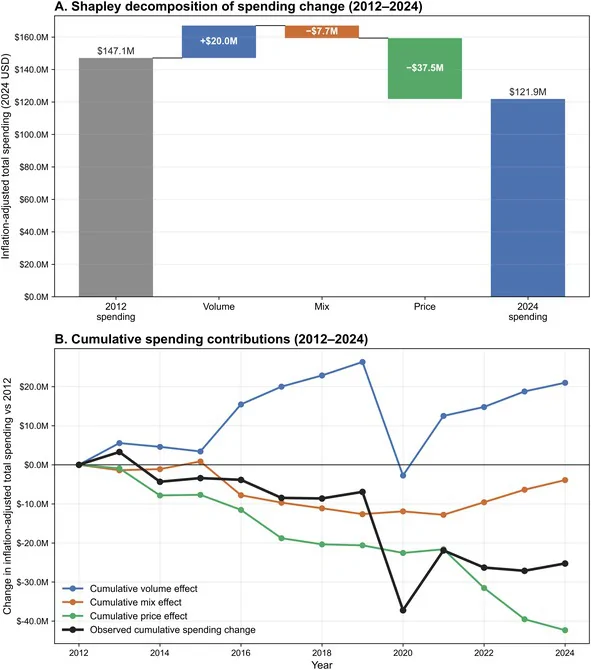

## 1 Introduction

Otologic and neurotologic procedures range from routine outpatient interventions to complex skull base surgeries, and are performed in both ambulatory and inpatient settings [1, 2]. As healthcare systems shift toward value‐based care, understanding the economic and Medicare reimbursement patterns surrounding these procedures has become increasingly important [3, 4]. Despite wide variation in technical complexity, few studies have examined longitudinal Medicare reimbursement at the procedure and provider level.

Medicare, the largest payer of healthcare services, reimburses physicians using the Resource‐Based Relative Value Scale (RBRVS), which assigns each CPT code a value based on physician work, practice expense, and malpractice cost [5, 6]. These relative value units (RVUs) are adjusted for geographic cost variation using the Geographic Practice Cost Index (GPCI) and multiplied by a national conversion factor, updated annually by the Centers for Medicare and Medicaid Services (CMS), to determine the final payment per service [7, 8]. Despite standardization, this system has been criticized for failing to keep pace with inflation and procedural complexity [9, 10].

In the field of otolaryngology, and otology/neurotology in particular, this reimbursement structure has important implications [11]. Many procedures, such as cochlear implantation, complex tympanomastoidectomy, and skull base approaches for tumor resection, require specialized equipment and extended operative time, yet their assigned RVUs may not reflect the technical demands nor resource utilization involved [12, 13]. Moreover, the growing emphasis on outpatient care, bundled payments, and cost containment has created additional financial pressures on high‐skill, low‐volume surgical fields [14, 15]. Against the backdrop of rising healthcare costs and fluctuating federal payment policies, the CMS reimbursement schedule provides a unique lens through which long‐term economic trends in otologic/neurotologic procedures can be analyzed.

Unlike prior reimbursement studies that examine individual procedures or limited procedural subsets, the following study analyzes a full range of otologic/neurotologic procedures and applies clustering methods to identify groups of procedures with similar economic behavior [3, 16]. Regional and temporal trends in procedure volume, payment volatility, and reimbursement erosion may disproportionately affect otologic/neurotologic procedures, yet these dynamics have not been comprehensively studied. Thus, we performed a 13‐year spatiotemporal analysis of Medicare reimbursement for otologic/neurotologic procedures to (1) characterize national trends in procedural volume, spending, and inflation‐adjusted reimbursement; (2) quantify the relative contributions of service volume, procedural mix, and within‐code reimbursement to aggregate spending change; (3) assess geographic variation in inflation‐adjusted payments; and (4) identify current procedural terminology (CPT) code clusters with similar economic profiles using unsupervised learning.

## 2 Methods

### 2.1 Data Source

This retrospective cross‐sectional study analyzed Medicare Part B reimbursement data for otologic/neurotologic procedures from 2012 to 2024. Data were obtained from the publicly available CMS Part B National Summary Data and Medicare Physician and Other Practitioner Files. CPT codes representing otologic, neurotologic, and skull base procedures with at least 100 total services were included, consistent with CMS reporting thresholds. Cochlear implant procedures were included in national trend analyses but excluded from clustering analyses because their reimbursement magnitude and device‐associated cost structure produced a dominant outlier cluster.

### 2.2 Variables and Preprocessing

The primary outcome was average inflation‐adjusted Medicare reimbursement per service for each CPT code per year. Secondary variables included annual service volume, total Medicare payment, coefficient of variation (CV) for reimbursement and volume, and compound annual growth rate (CAGR) across the study period. Financial variables were adjusted to 2024 U.S. dollars using the Consumer Price Index. Geographic reimbursement rates were extracted and mapped at the state and county level.

### 2.3 Outcomes and Analysis

Temporal trends in national otologic and neurotologic procedural volume, aggregate Medicare spending, and average per‐service payment from 2012 to 2024 were analyzed. Time‐series plots were created for the overall cohort and for the five most commonly billed CPT codes. The impact of the COVID‐19 pandemic was annotated in figures. State‐level reimbursement values were mapped, and percentage change in average per‐procedure reimbursement between 2013 and 2023 was calculated.

### 2.4 Spending Decomposition

To quantify drivers of aggregate spending change, inflation‐adjusted total Medicare spending was decomposed into contributions from overall procedural volume, procedural mix, and within‐code reimbursement. For each year *t*, total spending was expressed as $S_{t}=\sum_{i}q_{\mathrm{it}}p_{\mathrm{it}}=Q_{t}\sum_{i}\omega_{\mathrm{it}}p_{\mathrm{it}},$ where $q_{\mathrm{it}}$ denotes CPT‐specific service volume, $p_{\mathrm{it}}$ inflation‐adjusted payment per service, $Q_{t}$ total annual otologic/neurotologic procedural volume, and $\omega_{\mathrm{it}}$ each CPT code's share of annual volume. A Shapley decomposition framework estimated the contribution of each component to the change in inflation‐adjusted total spending between 2012 and 2024 by averaging across all possible factor‐order permutations. Year‐to‐year chain‐linked Shapley decompositions were cumulatively summed across the study period. A sensitivity analysis restricted decomposition to CPT codes present in both 2012 and 2024.

### 2.5 Unsupervised Learning and Clustering

To identify procedural groupings based on economic behavior, clustering was performed using four features: reimbursement CAGR, reimbursement CV, volume CV, and cumulative service count. Principal component analysis was used for dimensionality reduction and visualization. Clustering was performed using hierarchical agglomerative clustering and *k*‐means (*k* = 3 after cochlear implant exclusion). A three‐cluster solution was selected based on elbow plot inspection, cluster stability, and interpretability.

Analyses were performed in Python 3.11. No patient‐level data were used; the study was IRB‐exempt.

## 3 Results

### 3.1 Temporal Trends in Reimbursement and Service Volume

**FIGURE 1:**
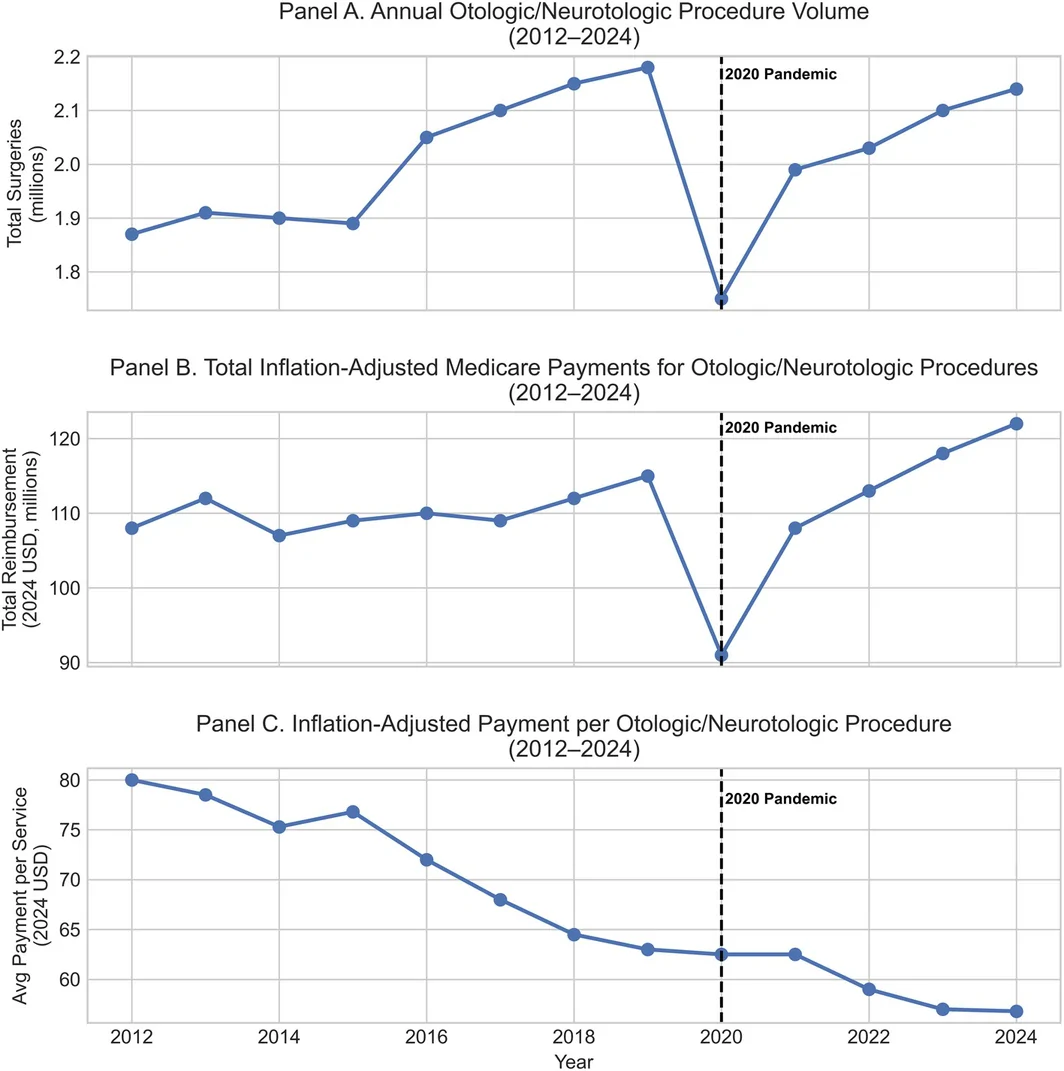
National trends in Medicare reimbursement for otologic/neurotologic procedures, 2012–2024. (A) Annual otologic/neurotologic procedural volume. (B) Total inflation‐adjusted Medicare payments, 2024 USD. (C) Inflation‐adjusted mean payment per procedure, 2024 USD. Dashed line marks the 2020 COVID‐19 pandemic.

Between 2012 and 2024, annual Medicare Part B–reimbursed otologic/neurotologic procedural volume increased from 1.85 million to 2.15 million services, a 16.2% increase (*p* = 0.002; Figure 1A). In contrast, total national Medicare spending did not demonstrate a significant temporal trend (*p* = 0.27; Figure 1B), reflecting opposing effects of rising volume and declining per‐service reimbursement. Inflation‐adjusted mean reimbursement per procedure declined from $80.47 in 2012 to $57.07 in 2024, a 29.1% reduction (*p* < 0.001; Figure 1C).

**FIGURE 2:**
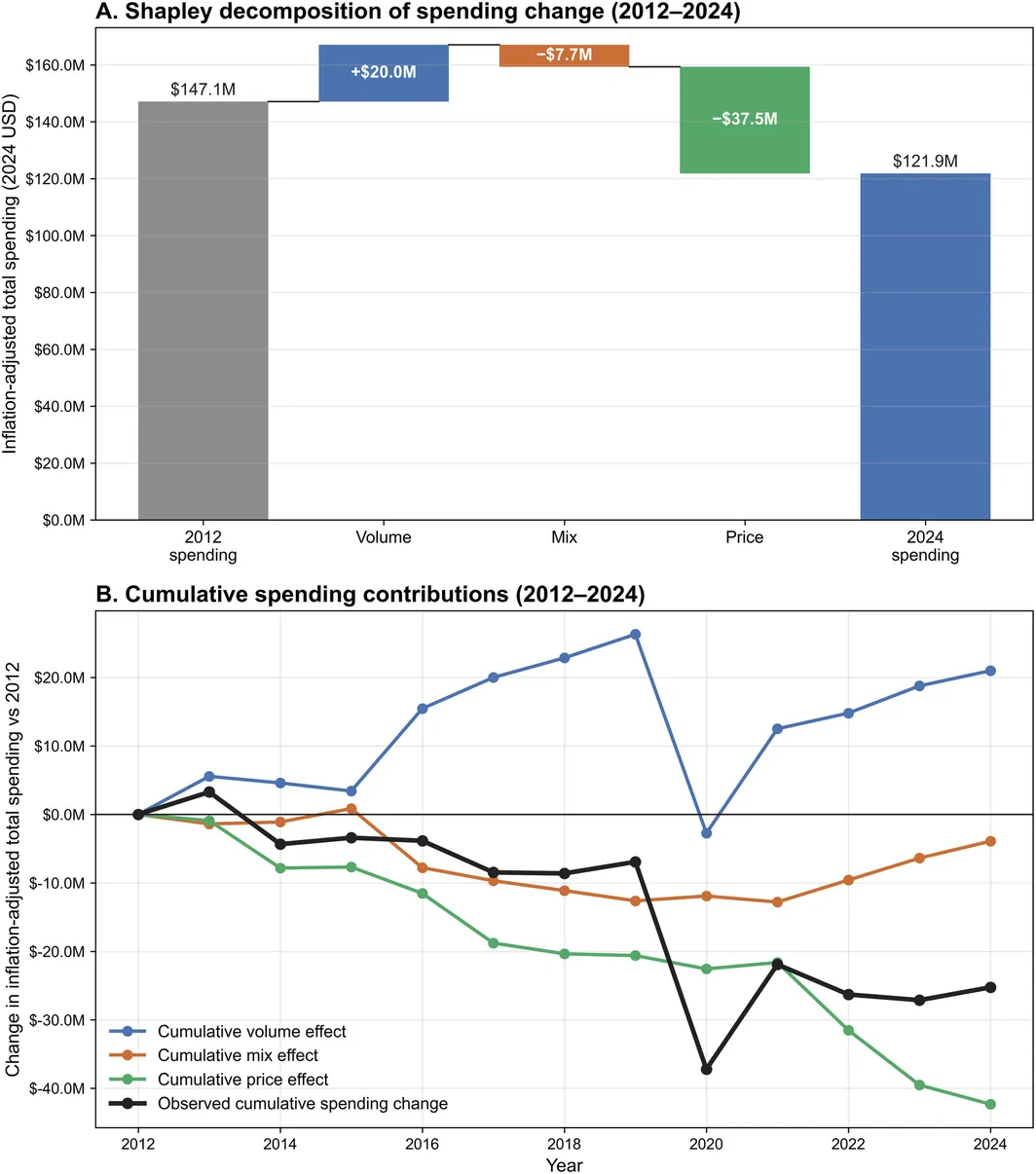
Spending decomposition of otologic/neurotologic Medicare reimbursement, 2012–2024. (A) Shapley decomposition of the change in inflation‐adjusted total spending into contributions from service volume, procedural mix, and within‐code reimbursement. (B) Cumulative yearly contributions of each component relative to 2012.

Shapley decomposition demonstrated that the 2012‐to‐2024 decline in otologic and neurotologic spending reflected offsetting effects of service volume, procedural mix, and within‐code reimbursement. Total spending declined from $147.1 million in 2012 to $121.9 million in 2024, a net decrease of $25.3 million (−17.2%). Increased total service volume exerted upward pressure on spending (+$20.0 million), whereas changing procedural mix contributed an additional downward effect (−$7.7 million). Declining inflation‐adjusted reimbursement per service was the dominant driver of reduced spending (−$37.5 million). In sensitivity analysis restricted to CPT codes present in both 2012 and 2024, the spending decline was larger (−$39.2 million) and was driven predominantly by reimbursement erosion (−$43.8 million), with minimal contribution from volume (+$1.2 million) and a small positive contribution from procedural mix (+$3.5 million) (Figure 2).

**FIGURE 3:**
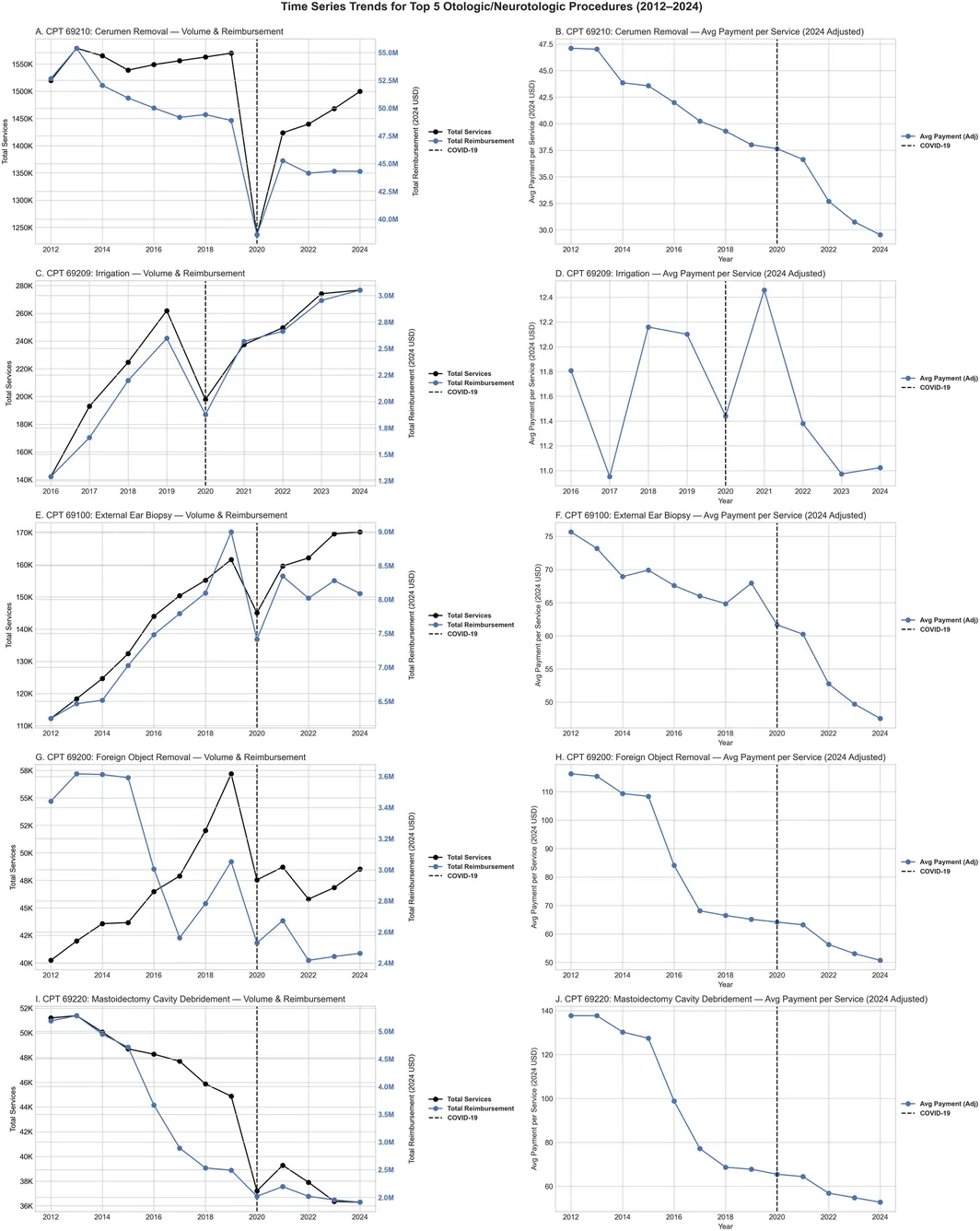
Time‐series trends for the five most frequently billed otologic/neurotologic CPT codes, 2012–2024. Panels show annual service volume and total inflation‐adjusted reimbursement, 2024 USD (A, C, E, G, I) and inflation‐adjusted payment per service, 2024 USD (B, D, F, H, J) for CPT 69210, 69209, 69100, 69200, and 69220. Dashed lines mark the 2020 COVID‐19 pandemic.

Procedure‐level analyses of the five most frequently billed CPT codes, which comprised 94.6% of total billing volume (range 93.8%–95.7%), revealed divergent trajectories in reimbursement and utilization (Figure 3). CPT 69210 remained the highest‐volume procedure, with steadily rising or plateauing annual services while inflation‐adjusted reimbursement per service declined from $47.37 to just under $30. Other high‐volume outpatient codes (69,209, 69,200, 69,100, 69,220) demonstrated similar reimbursement declines with modest variation in volume.

### 3.2 Geographic Variation in Reimbursement

**FIGURE 4:**
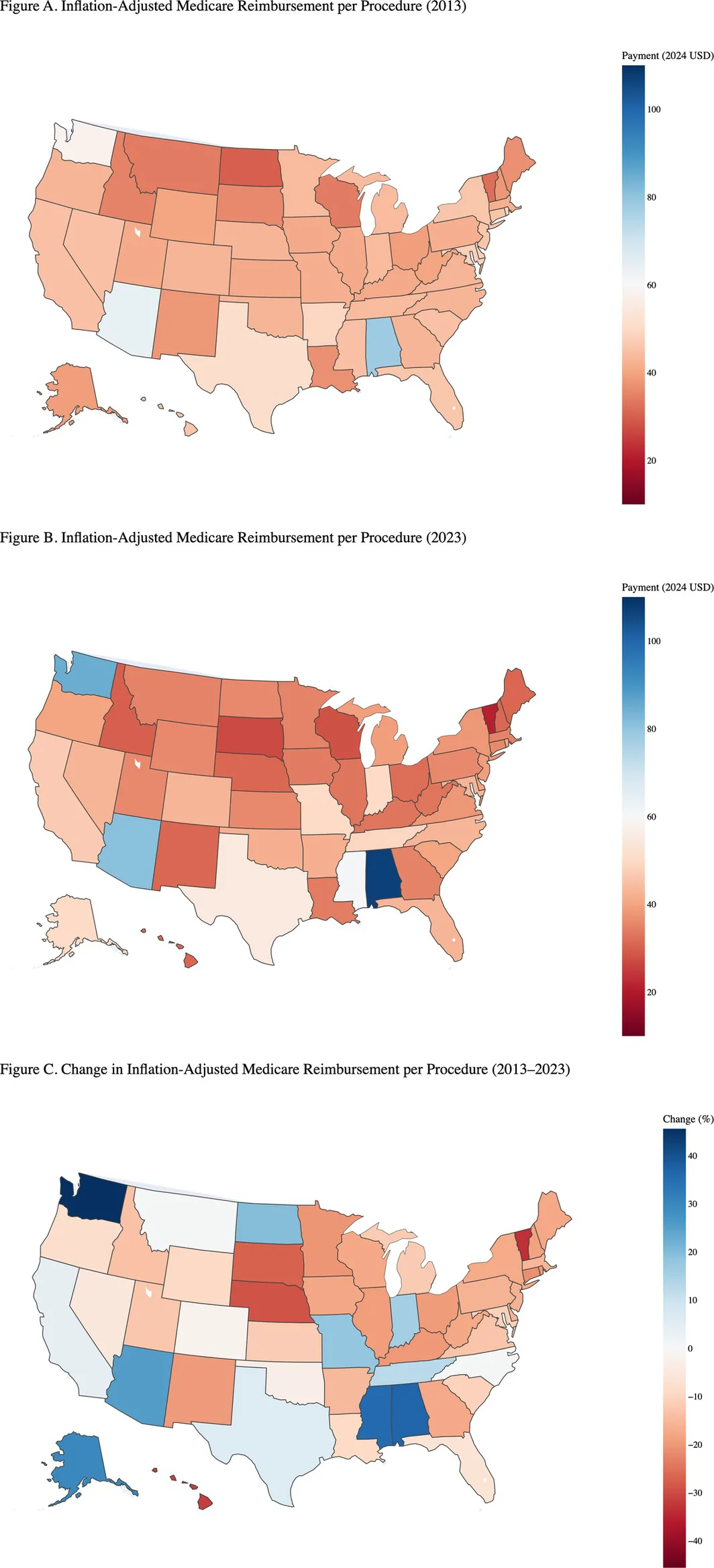
Geographic variation in inflation‐adjusted Medicare reimbursement for otologic/neurotologic procedures. (A) State‐level inflation‐adjusted reimbursement per procedure in 2013. (B) State‐level inflation‐adjusted reimbursement per procedure in 2023. (C) Percent change in mean inflation‐adjusted reimbursement per procedure from 2013 to 2023.

State‐level mapping demonstrated widening geographic variation in average inflation‐adjusted reimbursement per otologic/neurotologic procedure (Figure 4A–C). In 2013, reimbursement levels were generally higher across the Mountain West and northern Midwest, whereas several Southeastern states, particularly Alabama, were among the lowest‐reimbursed regions. By 2023, most states experienced inflation‐adjusted declines compared with 2013, including decreases exceeding 20% across portions of the Midwest, Mountain West, and Northeast. In contrast, a subset of states, including Alabama, Alaska, and Washington, demonstrated increases exceeding 40%. Alaska, California, and Washington consistently remained among the highest‐reimbursed states, whereas many Northeastern and Upper Midwest states remained on the lower end of the payment distribution.

**FIGURE 5:**
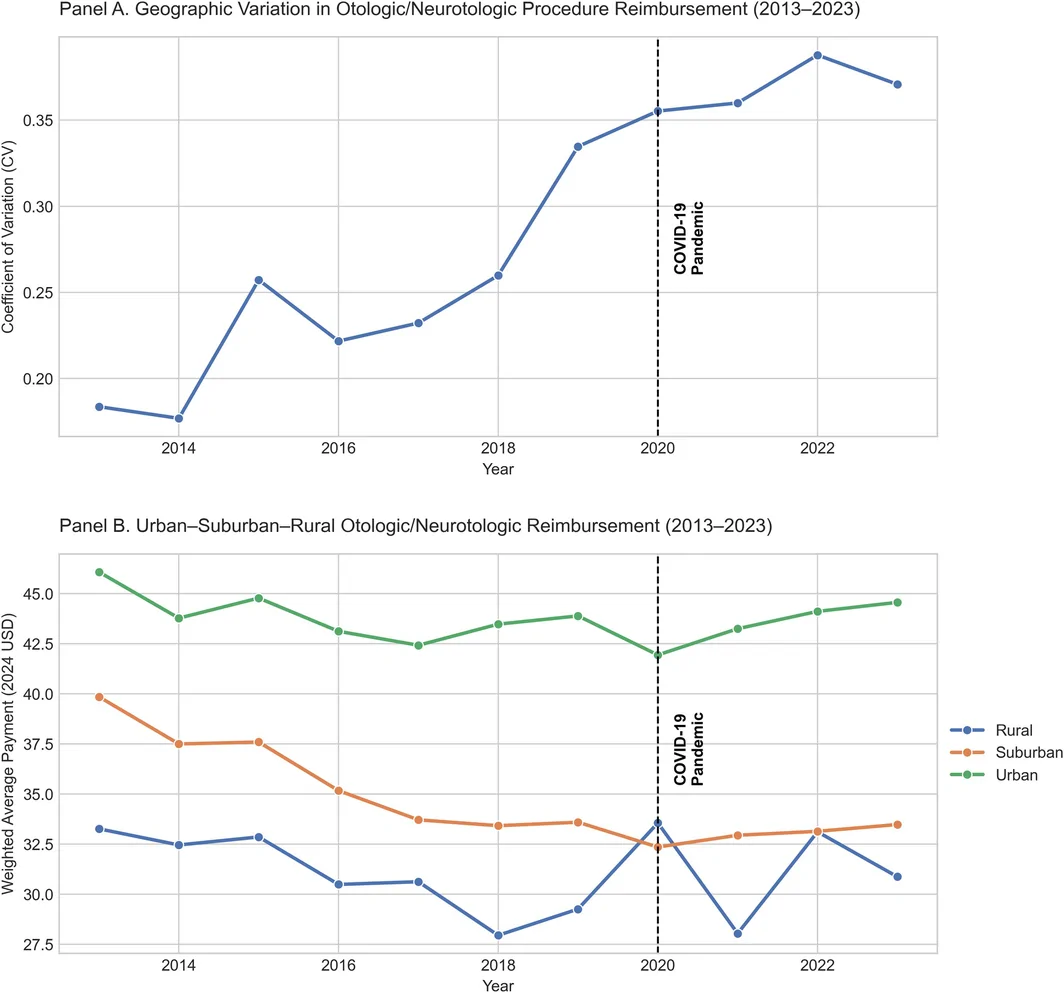
Geographic and rural–urban variation in otologic/neurotologic Medicare reimbursement, 2013–2023. (A) Temporal change in geographic variability of reimbursement across states, measured by coefficient of variation. (B) Weighted mean reimbursement by RUCA category. Dashed lines mark the COVID‐19 pandemic.

RUCA‐stratified analysis demonstrated rising geographic variability in reimbursement, with the CV increasing from 0.19 in 2013 to 0.37 after the pandemic (Figure 5A). Reimbursement levels also diverged by location type: rural providers consistently received the lowest average payments, while urban providers received the highest (Figure 5B). Mean urban reimbursement exceeded rural reimbursement by $11.90 (*p* < 0.0001), and this gap widened over time. Suburban regions, despite starting higher, declined more steeply than either urban or rural areas.

### 3.3 Clustering of CPT Codes by Economic Behavior

**FIGURE 6:**
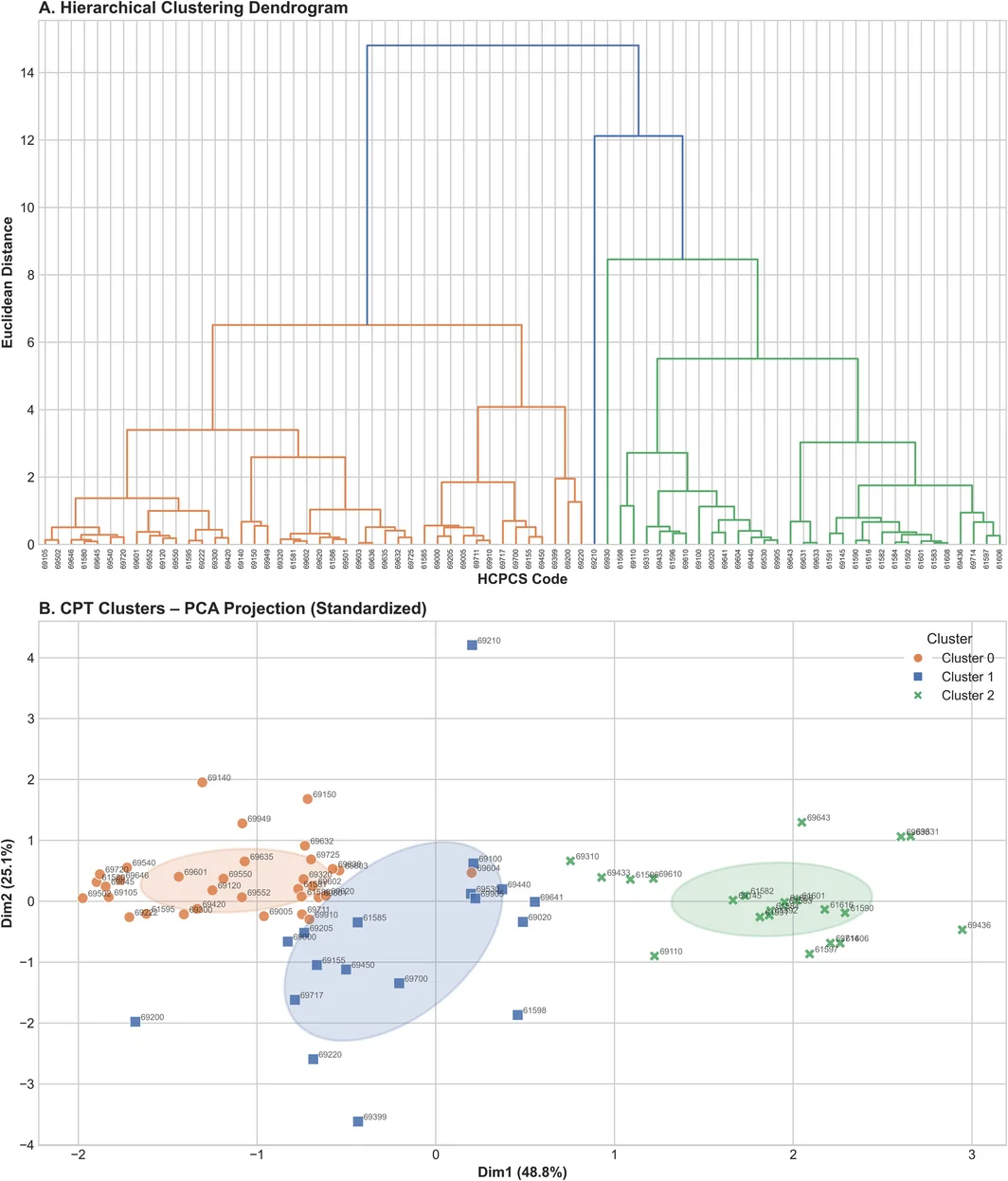
Unsupervised clustering of otologic/neurotologic CPT codes by economic behavior. (A) Hierarchical clustering dendrogram based on reimbursement growth, reimbursement variability, volume variability, and cumulative service volume. (B) Principal component analysis projection of the 3‐cluster solution after cochlear implant exclusion.

Unsupervised clustering initially identified four distinct groups of otologic/neurotologic CPT codes based on economic metrics: reimbursement volatility, volume variability, CAGR, and cumulative service count (Figure 6A)(Table S1). Cochlear implant procedures clustered into a single dominant group and were thus excluded from the clustering and PCA analyses to avoid disproportionate economic skew. PCA demonstrated partial cluster separation, with the first component capturing most variance in reimbursement magnitude and volatility (Figure 6B).

**FIGURE 7:**
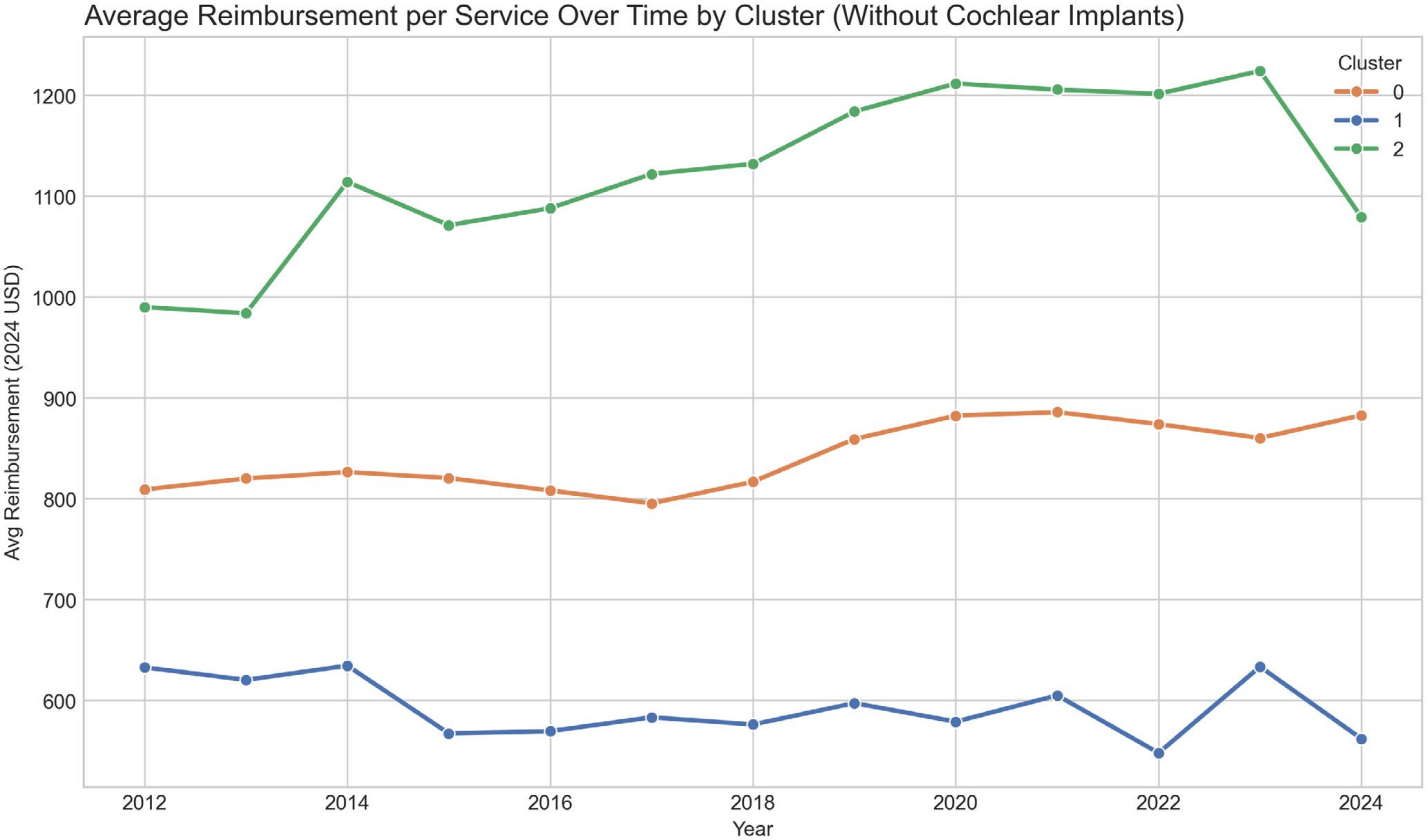
Longitudinal reimbursement trajectories by economic cluster. Mean inflation‐adjusted reimbursement per service over time for each otologic/neurotologic CPT cluster after cochlear implant exclusion.

Longitudinal reimbursement trajectories by cluster highlighted distinct economic patterns (Figure 7). Cluster 2, which included the highest‐cost procedures, demonstrated initial growth followed by stabilization between 2019–2023 and a sharp decline in 2024, reflecting heightened reimbursement volatility. Cluster 0 maintained intermediate reimbursement levels with relatively stable trends over time and minor year‐to‐year variation. Cluster 1, comprising the lowest‐cost outpatient procedures, exhibited the lowest average reimbursement across all years, with a modest decline and episodic fluctuations, particularly in 2016 and 2023.

**FIGURE 8:**
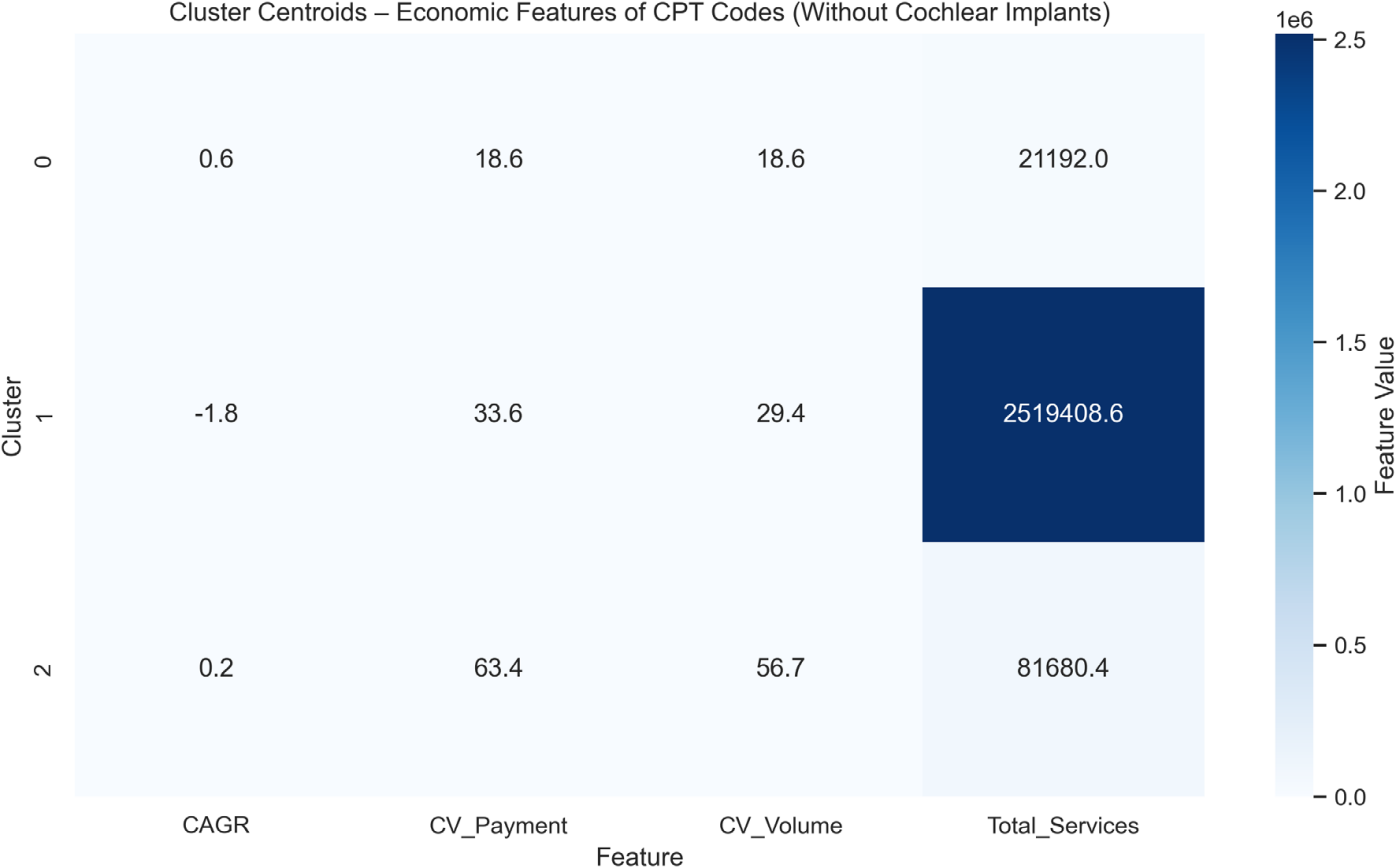
Cluster centroids of economic features for otologic/neurotologic CPT codes. Heatmap of cluster‐level mean reimbursement compound annual growth rate, reimbursement variability, volume variability, and cumulative service volume after cochlear implant exclusion. Reimbursement‐derived features were calculated from inflation‐adjusted 2024 USD values.

Cluster‐level centroids further quantified these economic distinctions (Figure 8). Cluster 1, which included the most frequently billed outpatient procedures, had the highest cumulative service volume (2.5 million) but demonstrated a negative CAGR = −1.8% and moderate payment variability (CV = 33.6). In contrast, Cluster 2 included high‐cost and high‐volatility procedures with elevated reimbursement CV (63.4) and volume CV (56.7), though total volume remained comparatively low (81,680 services). Cluster 0 occupied an intermediate position, with modest positive reimbursement growth (CAGR = 0.6), lower variability, and the smallest total volume (21,192 services). These quantitative profiles reinforce the procedural and financial divergence across clusters, with Cluster 1 representing cost‐efficient but declining reimbursement services and Cluster 2 comprising reimbursement‐sensitive, complex surgeries.

### 3.4 Clinical and Procedural Composition of Economic Clusters

Cluster‐wise composition by clinical category revealed meaningful differences in procedural intent and reimbursement structure (Figure S1). Cluster 1 was dominated by office‐based outpatient procedures, including cerumen removal and foreign body extraction. Cluster 2 included lower‐volume, higher‐complexity middle and inner ear surgeries such as tympanoplasty and labyrinthectomy. Cluster 0 contained a heterogeneous mix of infrequently billed specialized procedures, including temporal bone decompressions and unlisted codes. Despite lower absolute volume, Cluster 2 procedures accounted for a disproportionate share of total Medicare otologic/neurotologic expenditures because of their higher per‐service cost.

## 4 Discussion

This national study of Medicare Part B reimbursement for otologic/neurotologic procedures over 13 years demonstrates consistent reimbursement erosion despite rising procedural volume. Between 2012 and 2024, annual otologic/neurotologic procedural counts increased by more than 16%, yet inflation‐adjusted payment per service declined by 29.1%. Geographic variation persisted throughout the study period, and clustering identified three reproducible economic phenotypes among otologic/neurotologic CPT codes: high‐volume low‐cost services with reimbursement decline, high‐cost low‐volume procedures with volatile payment trends, and low‐volume stable‐cost codes. Together, these findings demonstrate an evolving economic landscape in otology and neurotology in which reimbursement is increasingly disconnected from clinical volume, technical complexity, and geographic equity.

### 4.1 National Volume and Reimbursement Trends: A Zero‐Sum Game

The increase in otologic/neurotologic procedural volume likely reflects both demographic pressures and shifts toward outpatient care. However, the 29% decline in inflation‐adjusted per‐service payment suggests that rising utilization has not translated into proportional financial support. Importantly, even nominally stable Medicare payments represent a real reimbursement cut when they fail to keep pace with inflation, because the purchasing power of each reimbursed service declines over time. This pattern is consistent with downward pressure from CMS's budget‐neutral conversion factor and periodic RVU revaluation. Prior studies show that procedural specialties have experienced steep reimbursement declines driven by conversion factor cuts and volume shifts [17, 18, 19]. This is particularly evident in Cluster 1, which includes procedures that dominate national volume yet demonstrate sustained negative reimbursement growth. These trends imply that even essential, low‐complexity services may become financially unsustainable when subjected to repeated annual cuts [3, 16, 20, 21, 22, 23, 24, 25, 26, 27, 28, 29, 30, 31, 32, 33, 34].

Formal spending decomposition further showed that stable or declining aggregate spending did not reflect stagnant demand, but rather a balance between increasing volume and persistent reimbursement erosion. In the full otologic/neurotologic CPT set, rising service volume partially offset spending losses, but this was more than counterbalanced by declining inflation‐adjusted within‐code reimbursement and a smaller adverse shift in procedural mix. When restricted to CPT codes present in both 2012 and 2024, reimbursement erosion accounted for nearly the entire decline in spending, reinforcing that the dominant long‐term driver was not reduced utilization but falling real payment per service. Year‐by‐year decomposition further suggested that the 2020 spending disruption was largely volume‐mediated, whereas the persistent post‐pandemic drag on spending was driven primarily by continued reimbursement decline.

### 4.2 Geographic Reimbursement Patterns and RUCA Disparities

The state‐level mapping revealed substantial variation in otologic/neurotologic reimbursement trajectories over time. From 2013 to 2023, multiple states in the Midwest and Northeast experienced > 20% declines in inflation‐adjusted payments, whereas states like Alaska, Alabama, and Washington exhibited net increases, highlighting growing regional disparities. In states such as Alabama, which were among the lowest‐reimbursed regions early in the study period, observed payment increases may alternatively reflect partial correction of a prior geographic undervaluation rather than true relative reimbursement growth. The stability of relative state reimbursement rankings over time suggests that observed geographic differences largely reflect within‐procedure payment variation rather than shifts in procedural mix. These patterns may reflect GPCI adjustment, regional supply–demand dynamics, or facility‐level factors. Prior analyses in neurosurgery, orthopedic surgery, otolaryngology, and various other specialties have demonstrated that geographic reimbursement gaps can influence physician distribution, practice viability, and patient access—particularly in under‐resourced settings [35, 36, 37, 38, 39, 40, 41, 42, 43]. As otolaryngology shifts further into outpatient and ambulatory domains, state‐level payment differences may influence whether lower‐reimbursed services remain accessible across all regions.

Despite a national fee schedule, RUCA stratification demonstrates that reimbursement inequities persist at the community level, with urban providers consistently receiving higher payments than rural providers over time, mirroring national access disparities already observed in otolaryngology [41, 44, 45]. This rural–urban reimbursement gradient likely reflects within‐procedure payment differences driven by GPCI‐based geographic adjustment and practice environment effects, rather than differences in procedural mix. Urban providers are more likely to practice in hospital‐affiliated or resource‐rich settings that influence billing patterns, with post‐pandemic consolidation amplifying preexisting disparities. Given that otologic and neurotologic services rely on specialized equipment and surgical expertise concentrated in urban centers, sustained reimbursement disadvantages in rural and suburban settings may further limit access to essential care for geographically vulnerable populations.

### 4.3 Economic Phenotypes and the Impact of Cochlear Implants

The distinct economic phenotypes identified by clustering carry important implications for reimbursement policy and practice sustainability within otology/neurotology. The concentration of most national volume within Cluster 1 was paired with the steepest inflation‐adjusted payment erosion, suggesting that common office‐based interventions are disproportionately vulnerable under current fee‐schedule dynamics. Persistent negative growth in these services may strain community otolaryngology practices that rely on high‐volume outpatient procedures to remain financially viable. In contrast, Cluster 2 represents resource‐intensive surgeries, including skull base approaches; elevated volatility in reimbursement may hinder long‐term investment in specialized equipment and workforce development. Cochlear implant procedures, however, are economically distinct from other otologic and neurotologic surgeries due to their high per‐service physician reimbursement, device‐associated cost structures, and relatively low procedural volume, which together generate disproportionately large contributions to total Medicare payments and variance across economic metrics. The necessity of excluding cochlear implant codes from clustering further underscores that device‐driven cost structures and high unit reimbursement may justify separate valuation strategies. Together, these patterns indicate that reimbursement models grounded in procedural intensity alone without an appropriate buoy may inadequately support both the highest‐volume and highest‐complexity ends of the otologic/neurotologic spectrum.

### 4.4 Implications for Value‐Based Care and Policy Reform

CMS currently relies on episodic RVU revaluation and specialty‐driven review processes that may fail to capture longitudinal reimbursement erosion or volatility across related procedures. Cluster‐based analysis may help identify CPT codes with persistent negative growth despite high utilization or disproportionate volatility relative to volume and complexity, thereby complementing existing RUC‐based reassessment efforts.

These findings are directly relevant to Medicare payment policy and future value‐based care models in otolaryngology. Persistent reimbursement erosion in high‐volume services may disincentivize efficient outpatient interventions, while volatility in high‐cost complex surgeries may undermine investment in subspecialty programs requiring long‐term planning and specialized resources. As bundled payments, site‐neutral reimbursement, and outpatient migration expand, policymakers should consider revisiting RVU assignments for procedures that have not kept pace with inflation or technical advancement [46, 47, 48].

RUCA‐adjusted analyses further suggest that payment disparities may compound access inequities in rural regions, where otolaryngologic services are already limited. Although individual surgeons may not respond directly to national reimbursement trends, these patterns should inform specialty‐society advocacy, CMS rulemaking comments, and institutional service‐line decisions.

## 5 Limitations

This study has limitations inherent to Medicare Part B files, which exclude Medicare Advantage and commercially insured populations and therefore do not fully represent national otologic/neurotologic practice. Aggregate CPT‐level data preclude adjustment for case complexity, operative time, comorbidities, or facility‐level cost variation, as well as further decomposition of within‐code reimbursement erosion into RVU, conversion‐factor, GPCI, and inflationary components. Although clustering identified distinct economic phenotypes, results may be sensitive to feature selection and do not incorporate device pricing or downstream episode costs. Finally, causal inferences regarding policy drivers remain limited given the descriptive design and absence of direct CMS conversion‐factor or RVU policy decomposition.

## 6 Conclusion

In this national spatiotemporal analysis, inflation‐adjusted Medicare reimbursement per otologic/neurotologic service declined substantially over 13 years despite rising procedural volume. Geographic variation widened over time, and clustering identified economically distinct groups of otologic and neurotologic procedures, including high‐volume outpatient services with persistent reimbursement erosion and complex low‐volume procedures with greater volatility. Spending decomposition further showed that aggregate spending decline was driven primarily by reimbursement erosion rather than reduced utilization, with additional contribution from procedural mix. Together, these findings suggest that current fee‐schedule mechanisms incompletely reflect the economic realities of otologic and neurotologic care and may warrant targeted modernization to sustain access across the care spectrum.

## Funding

The authors have nothing to report.

## Conflicts of Interest

The authors declare no conflicts of interest.

## Supporting information


**Figure S1:** Clinical Composition of Economic Clusters. Heatmap showing the distribution of clinical CPT categories within each economic cluster after cochlear implant exclusion.

**Table S1:** CPT Codes and Descriptions by Cluster.

## Data Availability

The data that support the findings of this study are available in Medicare Physician and Other Practitioners Public Use Files at https://data.cms.gov/provider‐summary‐by‐type‐of‐service/medicare‐physician‐other‐practitioners. These data were derived from the following resources available in the public domain: Centers for Medicare and Medicaid, https://data.cms.gov/.
