# Supplementary material for: Spatiotemporal Medicare Reimbursement Trends and Economic Clusters in Otologic/Neurotologic Surgery
Source: Laryngoscope. 2026 Jun 5;136(10):4444–56. doi: 10.1002/lary.70652 (PMC13569690; doi:10.1002/lary.70652)
Supplement: Supplementary file 1 [file LARY-136-4444-s002.docx]

**Supplementary Table 1:** CPT Codes and Descriptions by Cluster

| **Cluster 0** | | **Cluster 1** | | **Cluster 2** | | **Excluded Cluster 3** | |
| --- | --- | --- | --- | --- | --- | --- | --- |
| 61580 | Skull Base – Anterior/Approach-Extradural | 61585 | Skull Base – Anterior/Orbitocranial Approach with Orbital Exenteration | 61582 | Skull Base – Anterior/Approach-Extradural Frontal Craniotomy | 69930 | Inner Ear – Cochlear Implant |
| 61581 | Skull Base – Anterior/Approach-Extradural with Maxillectomy/Orbital Exenteration | 61598 | Skull Base – Posterior/Approach Transpetrosal | 61583 | Skull Base – Anterior/Approach-Intradural Frontal Craniotomy |  |  |
| 61586 | Skull Base – Anterior/Bicoronal Transzygomatic Approach | 69000 | External Ear – Incision/Drainage | 61584 | Skull Base – Anterior/Orbitocranial Approach Extradural |  |  |
| 61595 | Skull Base – Posterior/Approach Transtemporal | 69020 | External Ear – Incision/Drainage | 61590 | Skull Base – Middle/Approach Infratemporal Preauricular |  |  |
| 69005 | External Ear – Incision/Drainage | 69100 | External Ear – Excision | 61591 | Skull Base – Middle/Approach Infratemporal Postauricular |  |  |
| 69105 | External Ear – Excision | 69155 | External Ear – Excision | 61592 | Skull Base – Middle/Approach Orbitocranial Zygomatic |  |  |
| 69120 | External Ear – Excision | 69200 | External Ear – Foreign Body/Wax Removal | 61596 | Skull Base – Posterior/Approach Transcochlear |  |  |
| 69140 | External Ear – Excision | 69205 | External Ear – Foreign Body/Wax Removal | 61597 | Skull Base – Posterior/Approach Transcondylar |  |  |
| 69150 | External Ear – Excision | 69210 | External Ear – Foreign Body/Wax Removal | 61601 | Skull Base – Anterior/Definitive Intradural Lesion |  |  |
| 69222 | External Ear – Foreign Body/Wax Removal | 69220 | External Ear – Foreign Body/Wax Removal | 61606 | Skull Base – Middle/Definitive Intradural Lesion |  |  |
| 69300 | External Ear – Reconstruction | 69399 | External Ear – Miscellaneous | 61608 | Skull Base – Middle/Definitive Intradural Parasellar/Clival Lesion |  |  |
| 69320 | External Ear – Reconstruction | 69440 | Middle Ear – Incision | 61616 | Skull Base – Posterior/Definitive Intradural Lesion |  |  |
| 69420 | Middle Ear – Incision | 69450 | Middle Ear – Incision | 69110 | External Ear – Excision |  |  |
| 69501 | Middle Ear – Excision | 69530 | Middle Ear – Excision | 69145 | External Ear – Excision |  |  |
| 69502 | Middle Ear – Excision | 69641 | Middle Ear – Tympanoplasty/Mastoidectomy | 69310 | External Ear – Reconstruction |  |  |
| 69540 | Middle Ear – Excision | 69700 | Middle Ear – Exploration | 69433 | Middle Ear – Incision |  |  |
| 69550 | Middle Ear – Excision | 69717 | Middle Ear – Auditory Osseointegrated Implant | 69436 | Middle Ear – Incision |  |  |
| 69552 | Middle Ear – Excision | 69905 | Inner Ear – Labyrinthectomy | 69610 | Middle Ear – Repair |  |  |
| 69601 | Middle Ear – Repair |  |  | 69631 | Middle Ear – Tympanoplasty/Mastoidectomy |  |  |
| 69602 | Middle Ear – Repair |  |  | 69633 | Middle Ear – Tympanoplasty/Mastoidectomy |  |  |
| 69603 | Middle Ear – Repair |  |  | 69643 | Middle Ear – Tympanoplasty/Mastoidectomy |  |  |
| 69604 | Middle Ear – Repair |  |  | 69714 | Middle Ear – Auditory Osseointegrated Implant |  |  |
| 69620 | Middle Ear – Repair |  |  |  |  |  |  |
| 69632 | Middle Ear – Tympanoplasty/Mastoidectomy |  |  |  |  |  |  |
| 69635 | Middle Ear – Tympanoplasty/Mastoidectomy |  |  |  |  |  |  |
| 69636 | Middle Ear – Tympanoplasty/Mastoidectomy |  |  |  |  |  |  |
| 69645 | Middle Ear – Tympanoplasty/Mastoidectomy |  |  |  |  |  |  |
| 69646 | Middle Ear – Tympanoplasty/Mastoidectomy |  |  |  |  |  |  |
| 69711 | Middle Ear – Auditory Osseointegrated Implant |  |  |  |  |  |  |
| 69720 | Inner Ear – Facial Nerve Decompression |  |  |  |  |  |  |
| 69725 | Inner Ear – Facial Nerve Decompression |  |  |  |  |  |  |
| 69910 | Inner Ear – Endolymphatic Shunt |  |  |  |  |  |  |
| 69949 | Unlisted Otologic Procedure |  |  |  |  |  |  |
